# Cigarette Smoking and Schizophrenia: Etiology, Clinical, Pharmacological, and Treatment Implications

**DOI:** 10.1155/2021/7698030

**Published:** 2021-12-13

**Authors:** Jack Baichao Ding, Kevin Hu

**Affiliations:** ^1^Division of Medicine, Royal Adelaide Hospital, SA Health, Adelaide, Australia; ^2^Division of Medicine, Lyell Mcewin Hospital, SA Health, Adelaide, Australia 1 McArthur Ave, Rostrevor, Adelaide, 5073, Australia

## Abstract

Recent data suggests that the prevalence of smoking in schizophrenia remains high. While reports suggest that smoking increases the risk of developing schizophrenia, the potential causative role of smoking in this relationship needs further investigation. Smokers with schizophrenia are more likely to have more intense positive symptoms and lower cognitive function, but diminished intensity of extrapyramidal side effects than nonsmoking patients with schizophrenia. They were also more likely to exhibit aggressive behaviour compared to nonsmokers, which could suggest higher levels of baseline aggression. The significant cost associated with regular tobacco expenditure can detract from investment in key domains. Large-scale trials have shown that pharmacotherapy for smoking cessation is effective and does not worsen the risk of developing neuropsychiatric symptoms compared to placebo. Electronic cigarette use among schizophrenia patients is high, and there is emerging evidence supportive of its efficacy. Future improvements include large-scale trials assessing the utility, efficacy, and safety of electronic cigarettes in schizophrenia patients.

## 1. Introduction and Background

### 1.1. Breadth of the Issue

The association between smoking and schizophrenia was first observed several decades ago [1]. Since then, an abundance of research has been published regarding the causes and consequences of smoking in schizophrenia. Smoking in psychotic illnesses is common, with its prevalence in schizophrenia being particularly high at around 70-80% [2]. The prevalence of cigarette smoking in the first episode psychosis was comparably high at an estimated 58%, with one meta-analysis reporting a strong association between the two (OR = 6.04; 95% CI, 3.03-12.02) [3]. In line with trends in the general population, male smokers engaged in smoking more frequently than females [4]. Smokers with schizophrenia inhaled deeper and for longer durations, compared to normal controls, thereby exposing themselves to higher levels of toxic elements of tobacco [5]. Additionally, they also consumed higher quantities of cigarettes and had higher levels of nicotine dependence compared to smokers without schizophrenia [6]. Low education, unemployment, severe negative symptoms, increased daily caffeine intake, and substance abuse have been linked to tobacco smoking in people with psychotic disorders [7]. Since the average life expectancy of individuals with schizophrenia is between 61 and 71 years, with cardiovascular disease accounting for as much as 50% of excess mortality in schizophrenia, the issue of tobacco smoking is therefore very relevant in terms of prognostic implications [8]. One study suggested that cigarette smoking increased the risk of mortality from cardiovascular disease in people with schizophrenia by 86% over a 20-year period, with the overall 20-year all-cause mortality risk being 30% [9].

### 1.2. Mechanism of Action of Nicotine

Nicotinic acetylcholine receptors (nAChRs) are protein receptors that normally interact with the endogenous neurotransmitter acetylcholine but can also be modulated by exogenous drugs such as nicotine ([10] : 1-46). There are two broad categories of nAChRs: muscle receptors and neuronal receptors. At the muscle level, nAChRs mediate the neuromuscular communication that drives muscle contraction, and at the neuronal level, they are involved in synaptic transmission ([10] : 1-46). The intrinsic ion channels of nAChRs are activated by nicotine binding and results in the movement of cations (Na+, Ca2+, and K+) across the cell membrane, which trigger voltage-gated calcium channels to open, and ultimately the release of neurotransmitters [11]. Notably, nicotine action on the nAChRs of neurons that project to the ventral tegmental area (VTA) and the mesolimbic pathway is thought to release dopamine, which occurs partly in relation to the activation of the reward circuitry of the VTA [12]. Along with cholinergic signalling in the nucleus accumbens, amygdala, and hippocampus, this action is thought to play a key role in the mechanism of nicotine addiction [13]. Nicotine has been shown to modulate the release of nearly all neurotransmitters, including dopamine, glutamate, noradrenaline, serotonin, opioids, acetylcholine, and *γ*-aminobutyric acid (GABA) [13].

## 2. Review

### 2.1. Etiology of Tobacco Use in Schizophrenia

**Figure 1:**
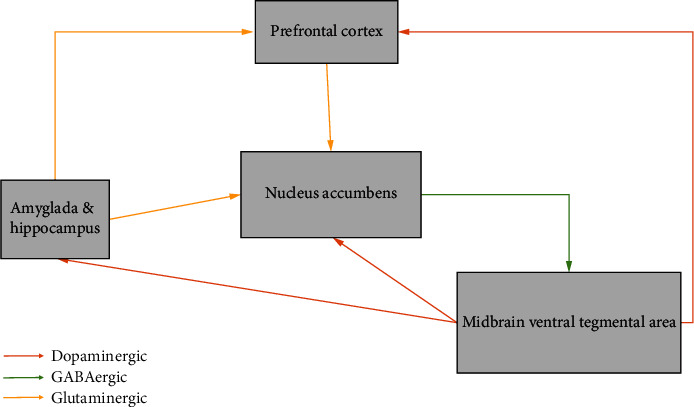
Diagram outlining key components of reward circuits of the midbrain. Nicotine administration activates midbrain dopaminergic pathways [20].

The underlying factors driving high smoking rates in patients with schizophrenia are unclear, though several hypotheses exist. One is a self-medicative hypothesis, which suggests that nicotine interacts with central nAChRs causing the release of dopamine and serotonin, thus resulting in stimulant effects secondary to the dopaminergic activity, driving the individual's desire to engage in smoking behaviour [14]. Further, nicotine has demonstrated significant inhibitory effects towards both monoamine oxidase (MAO) types A and B, which normally degrade dopamine into inactive metabolites [15]. Therefore, the net result is increased dopaminergic activity secondary to increased release and decreased degradation from smoking. This could function as a counterweight to hypofunction of central nAChRs [16], which is well documented in schizophrenic patients in the medical literature. This theory aligns with the dopamine hypothesis of the pathophysiological basis of schizophrenia, which stipulates those positive symptoms which result from a hyperactive mesolimbic pathway, whereas the negative symptoms arise from a hypoactive mesocortical pathway [17]. In the context of that pathway, it is hypothesized that schizophrenic patients would attempt to alleviate negative symptoms via nicotine, which increases dopamine and glutamate levels in the prefrontal cortex [16]. Indeed, there is research that suggests that people with schizophrenia who smoke may have fewer negative symptoms [18]. In addition, recent research also suggests that smoking can ameliorate the intensity of extrapyramidal symptoms arising from antipsychotic medication, which is evidence of another possible symptomatic motive for smoking in schizophrenia [19]. The underlying mechanism is thought to involve nicotine interacting with nAChRs in the VTA, thereby raising dopamine levels in the striatum through the mesolimbic dopaminergic pathway, which dampens the effect of dopamine receptor blocking antipsychotics and consequently lessens extrapyramidal symptoms [20]. A diagrammatic representation of this can be seen in Figure 1.

This hypothesis that negative symptoms in schizophrenia are alleviated by smoking is contradicted by several studies. One cross-sectional study of 131 schizophrenic patients revealed that patients with mild-moderate dependence, but not severe dependence, had greater scores on the Positive and Negative Syndrome Scale (PANSS), and smokers with mild nicotine dependence had greater negative symptoms compared to nonsmokers [21]. However, studies of this design are limited by the reliability of patient self-assessment of smoking status. Additionally, a limitation to the cross-sectional design of the study is that patients who smoke may have a predisposition to more negative symptoms at baseline than nonsmokers and that it is possible that cigarette use may in fact ameliorate negative symptoms in this distinct subgroup. Further research comparing negative symptoms during periods of abstinence and periods where individuals smoke (either prior to smoking or during sustained quit periods) would be needed to strengthen evidence for the hypothesis. A 2013 systematic review concluded that smoking was linked with increased psychiatric symptom severity in schizophrenia, in direct contradiction of the self-medication hypothesis [22].

An alternative theory is that tobacco smoking plays a role in the development of schizophrenia. This theory is in line with the high prevalence of smoking in patients with first-onset psychosis at 58% [3]. An Israeli cohort study of 14,248 military recruits without any major mental illness was followed over a period of 4 to 16 years. It was shown that participants who smoked over 10 cigarettes per day were 2.28 times more likely to develop schizophrenia (95%CI = 1.19-4.34) than those who did not [23, 24]. The main limitation to this study was that it did not adjust for cannabis use, a potential confounder. A larger prospective Swedish study of 233,879 males and 1,413,849 females without a history of psychosis adjusted for general drug use as a potential confounder and discovered elevated hazard ratios for the first-onset schizophrenia in both light smokers and heavy smokers [25]. Notably, the hazard ratio of heavy smoking was 3.45 for females and 3.80 for males, compared to ratios of 2.21 (females) and 2.15 (males) for light smokers, thus supporting the hypothesis that smoking causes schizophrenia. These findings are corroborated by a 2020 meta-analysis analysed 12 prospective studies and found an overall increased risk of developing schizophrenia in smokers versus nonsmokers (RR = 1.99, 95% CI: 1.10-3.61) [26]. A novel finding was that prenatal exposure to smoke increased the risk of schizophrenia by 29%. None of the studies adjusted specifically for cannabis use, which may potentially be a confounding factor unaccounted for.

A third theory is that there is a shared genetic propensity in smoking and the development of schizophrenia [27]. One study investigated genetic variants associated with tobacco use and schizophrenia with data extracted from the genome-wide association studies. Its findings suggested a causative effect of initiating tobacco use and developing schizophrenia (OR: 1.73, 95% CI: 1.30-2.25, *p* < 0.001). However, this finding was made nonsignificant after the impact of variants from other genes was accounted for (OR: 1.03, 95% CI: 0.97-1.09, *p* = 0.32) [28]. A large Icelandic study analysed 144, 609 participants without a history of psychosis and discovered higher polygenic risk scores for developing schizophrenia in people with any smoking history compared to people who never smoked [29]. These results are indicative of overlapping genetic roots for schizophrenia and smoking. The genetic propensity hypothesis is challenged by a Swedish study that contrasted the smoking habits of monozygotic female twins, biological sisters and half-sisters, and first-degree female cousins and the development of nonaffective psychosis. It reported a declining relationship between smoking and the development of schizophrenia in identical twins compared to relatives or progressively more distant relatives, or the general population. This suggested part of the comorbidity between smoking and schizophrenia may be attributable to genetic overlap, in support of the genetic risk hypothesis. The study also found that among relatives who smoked; the smoker was more likely to develop schizophrenia, in support of the hypothesis that smoking causes schizophrenia. Notably, in identical twins, the association between smoking and schizophrenia was not definitive [25]. There is no study showing that administration of nicotine leads to psychosis development; however, in contrast, there are also no studies showing that cannabis administration in healthy individuals precipitates psychosis. These findings suggest that genetic factors alone do not completely account for the positive association between smoking and development psychotic symptoms.

### 2.2. The Clinical and Social Implications of Smoking in Schizophrenic Patients

The link between smoking, psychiatric symptoms, and extrapyramidal side effects of antipsychotic medication in patients with schizophrenia has been controversial. A 2019 meta-analysis of 29 studies concluded that smoking caused no significant alterations in depressive, anxiety, and negative symptoms in smokers with schizophrenia and nonsmokers with schizophrenia. However, smoking was found to increase positive symptom intensity and alleviate extrapyramidal side effects [19]. These findings could be explained by the dopaminergic and stimulant effects of nicotine [14]. Another mechanism could potentially involve the CYP1A2 enzyme. In terms of the cognitive impact of smoking in schizophrenia, a 2020 meta-analysis of 18 studies noted significant deficits in attention, executive function, working memory, learning, reasoning, and processing in chronic smokers compared with nonsmokers [30]. This contrasts with acute administration of nicotine in a general population, where broad improvements in cognitive function have been demonstrated [31]. One explanation is that smokers with schizophrenia have lower cognitive function at baseline, compared to nonsmokers with schizophrenia. However, given the cross-sectional design of studies selected, causation could not be determined. The cognitive impairment associated with chronic smoking in schizophrenia is particularly relevant due to psychosocial functioning and interpersonal interactions being important prognostically.

One study of 474 schizophrenia patients assessed the relationship between aggressiveness and childhood trauma in schizophrenia and reported significantly higher physical aggression scores in smokers versus nonsmokers [32]. This finding is notable as studies have reported nicotine replacement to significantly negate agitation in smokers with schizophrenia in the past [33]. One possibility is that schizophrenia participants who smoke have a higher baseline level of agitation and aggression compared to nonsmokers and therefore smoke to alleviate the hostility. This theory would be supportive of the self-medication hypothesis. Indeed, one study investigated hostility in participants without a history of mental illness and reported that certain aggressive behaviours may be a risk factor initiating smoking, which could suggest that this phenomenon is not necessarily limited to smokers with schizophrenia [34].

Besides clinical symptoms, tobacco smoking in schizophrenia gives rise to significant social ramifications. One US study of 78 participants found that people with schizophrenia or schizoaffective disorder on public assistance spent around 27% of their monthly allowance on cigarettes [35]. Some have suggested that this disproportionate expenditure on cigarettes hinders the ability of schizophrenia patients to appropriately allocate funds to areas, such as food and social situations, in which their welfare would be promoted [36].

### 2.3. Pharmacological Implications

Cytochrome P450 (CYP) enzymes are a family of enzymes that are most concentrated in the liver. They are heavily involved in drug metabolism, accounting for up to 75% of the process [37]. The CYP1A2 member is of relevance in schizophrenia patients, as it plays a significant role in the metabolism of both clozapine and olanzapine [38, 39]. Polycyclic aromatic hydrocarbons generated from tobacco smoking have been shown to increase the activity of CYP1A2, thus increasing the metabolism of olanzapine and clozapine, which ultimately results in decreased plasma concentrations of the drugs [40]. This is important in clinical practice, because without dose adjustments, smokers may be at risk of subtherapeutic psychopharmacological management. A 2014 meta-analysis of seven studies suggested lowering olanzapine and clozapine doses by 30% and 50%, respectively, in nonsmoking schizophrenia patients in comparison to smokers [41]. Interestingly, caffeine is also metabolized by CYP1A2, and studies have found smoking increases caffeine clearance by inducing CYP1A2 activity, as it does with clozapine and olanzapine [42]. One meta-analysis found that cigarette smoking of at least five cigarettes a day and consuming more than three cups of coffee a day was associated with diminished efficacy but increased safety of olanzapine [43]. In the context of smoking cessation, it has been suggested that lowering doses of clozapine and olanzapine by 30-40% was necessary to restore serum concentrations to previously stable levels [44].

### 2.4. Smoking Cessation in Schizophrenia

One calculation estimated that people with schizophrenia die around 28 years earlier than people without a psychiatric history, with tobacco smoking-related diseases directly contributing to the bulk of the risk [45]. Another study analysed 174,277 patients with schizophrenia who were hospitalized in California from 1990 to 2005 and concluded that around 53% of total deaths since initial admission were attributable to a disease that was causally linked to tobacco use [46]. An aspect of concern with smoking cessation in schizophrenia is the potential weight gain and its contribution to early mortality. One study noted an average weight gain of 4.8 kg in schizophrenia patients who had ceased smoking over a 12-month period, versus 1.2 kg gain in those who had relapsed over the same period. Notably, the abstinent group demonstrated a 7.6% reduction in Framingham risk score, suggesting that smoking cessation can reduce 10-year cardiovascular mortality risk despite the additional weight gain [47].

The first line pharmacologic options for smoking cessation among members of the general population include varenicline, bupropion, and nicotine replacement therapy (NRT). Varenicline monotherapy and combination NRT have the highest abstinence rates, while bupropion and single NRT are superior to placebo [48]. In the schizophrenia demographic, a 2020 meta-analysis concluded that varenicline, bupropion, and NRT were all superior to placebo, with varenicline also being superior to bupropion (RR: 2.02, 95% CI: 1.04-3.93; *p* = −0.038). None of the medications were associated with increased psychiatric manifestations, though varenicline did pose an increased risk of nausea. The measurable effectiveness of all three medications was low, with varenicline at 22%, bupropion at 18%, and NRT at 9% [49]. The primary limitation of this meta-analysis was a deficiency of high-quality studies of longer than 12 weeks duration. One study randomized patients after 12 weeks of open-label treatment with varenicline to placebo or continued use of varenicline and found substantial increases in quit rates in patients continued on varenicline [50]. Therefore, the endpoints of each medication may not be representative of the treatment intention of long-term abstinence. In addition, there is a paucity of data on patients with active psychiatric symptoms or polysubstance use disorder. While the evidence suggests that pharmacotherapy is likely to be more useful than placebo for attaining smoking cessation in schizophrenia patients, they are less likely to receive medical management compared to smokers without mental illnesses [51]. A novel finding is that the psychiatric population has a low success rate of smoking cessation (around 4%) across multiple trials [52, 53]. This suggests that behavioural interventions alone are likely ineffective for schizophrenia patients. One possible explanation for this is organic neurologic abnormalities observed in these patients, including abnormalities in nicotinic acetylcholine receptor function and regulation [54].

Case reports of worsening neuropsychiatric symptoms in smokers with mental illnesses being treated with varenicline and bupropion gave rise to the EAGLES trial, in which varenicline, bupropion, and NRT were compared for efficacy and safety in groups with and without psychiatric illnesses. There were around 8000 participants in total, of which 390 had schizophrenia or schizoaffective disorder. The rate of patients with mental illnesses suffering moderate or severe neuropsychiatric adverse events was reported to be 6.5% for varenicline users, 6.7% for bupropion users, 5.2% for NRT users, and 4.9% for placebo users. Overall, the study concluded there was no significant increase in moderate to severe neuropsychiatric adverse events in any of the three drugs in the psychiatric population when compared to placebo. The primary limitations of the study included an endpoint of 24 weeks and the use of smokers who were psychiatrically and medically stable. Therefore, the efficacy findings may not be representative of clinical conditions and may not be generalizable to patients with obvious psychiatric symptoms [52].

### 2.5. Electronic Cigarette Smoking and Schizophrenia

Electronic cigarettes (e-cigarette) that deliver nicotine without tobacco were first introduced on the market around 2006 [55]. Since then, its use has risen significantly, with a recent study reporting a prevalence of 9.2% in university students in the previous 12-month period. Notably, the study also found that electronic cigarette usage was associated with psychiatric disorders and impulsive traits [56]. Rates of electronic cigarette consumption were similarly high among schizophrenia patients, with 7% current users, 37% previous users, and 24% of the remaining never users open to trying them. Notably, 37% of schizophrenia patients smoked electronic cigarettes with the intention to quit tobacco smoking [57]. Indeed, one study suggested that electronic cigarette use in patients with schizophrenia caused a 50% reduction in daily tobacco cigarette consumption in 50% of participants (from 30 cigs/day to 15), whereas 14.3% of participants had quit altogether by week 52. The primary limitation of this study was its small sample size of 14 subjects and a study design that lacked a placebo arm [58]. Thus far, there are only three case reports on the impact electronic cigarettes have on clozapine levels [59–61]. Importantly, the CYP1A2 enzyme is thought to be altered by hydrocarbons in cigarette smoke, rather than nicotine content, which can be delivered by both tobacco and electronic cigarettes [59]. Given the lack of data, there is currently no recommendation regarding dose adjustment of antipsychotics in the context of electronic cigarette use.

### 2.6. Role of Tobacco Industry

People with psychiatric disorders make up one of the largest groups of smokers and are responsible for up to 44% of cigarette sales in the United States [62]. Researchers with tobacco industry affiliations first studied the connection between psychiatric illnesses and smoking in the 1950s, at a time when nonindustry researchers were first signalling the possible association between smoking and cancer in healthy civilian populations. The tobacco-funded research of this time focused on the supposed lower rates of cancer in schizophrenia smokers, with some papers posing the question as to whether it was practical to quantify the association [1, 63]. However, the schizophrenia cancer statistics were often stratified by proportionate mortality, a number obtained by the cancer deaths divided by total death. This was a flawed calculation, as schizophrenia patients had a higher baseline total death due to various comorbidities [64]. The most prominent effect of this early research was the leading belief that patients with schizophrenia were less susceptible to cancer because of an organic mechanism, a concept that endured until the later 1980s [65].

In the 1980s, the tobacco industry began to fund and proliferate the self-medication hypothesis of schizophrenia and smoking [66]. This agenda was published through multiple outlets, such as the scientific literature and published print books. One example was a 1998 review article written by an industry-affiliated author that argued that nicotine may help patients help themselves and a proposal to reframe nicotine psychopharmacology as “therapeutic” rather than “addictive” [67]. Concluding statements of the article revolved around the potential of tobacco cigarettes to alleviate psychosomatic pain and symptoms of schizophrenia. While funding such studies helped advance the tobacco industry by diminishing the escalating criticism and hostility against cigarettes, some authors have argued that it may have also helped construct a foundational research framework and relations between corporate bodies and academic researchers and institutions [68]. Indeed, the self-medication hypothesis was and remains a legitimate hypothesis of smoking and schizophrenia, and tobacco industry funding did establish preliminary groundwork and arguments regarding it. Nevertheless, there are several concerns regarding corporate sponsorship and its potential negative effects on study design and research questions, with one study concluding that industry funding detracts research focus away from questions most relevant to public health [69].

In the late 1980s, researchers without ties to the tobacco industry gradually became increasingly critical of tobacco-affiliated investigators and their researchers. This culminated in the critical analysis of the role of the tobacco industry as one of the forefront funders of medical research and the negatives of this in the 1990s [70]. At this time, tobacco-funded researchers were heavily scrutinized, as were their research proposals and conclusions. In the early 2000s, the tobacco industry cultivated a new image and proposed transparency in its involvement in scientific research in the context of an increasingly critical public. Still, a major study of the time concluded that tobacco industry research revolved around projecting a positive corporate image above public health policies and that the escalation in transparency may be a part of this agenda [71]. Ultimately, in terms of the influence the smoking industry had on the scientific understanding of smoking and schizophrenia, some authors have argued that arguments towards the negative may be prone to exaggeration. One cited example is Hollywood documentary productions that influence public beliefs and applying contemporary ethical and scientific standards through a hypercritical retrospective lens, disregarding the normalities of the time [68]. After all, investigating and advancing viewpoints considered controversial or novel, compared to the mainstays of the time, are well within the realms of scientific ethics. In addition, obtaining funding for medical research at the time can be regarded as within public interest, despite the source of funding. In contrast, other authors have suggested that the tobacco industry had countered nonindustry research and promoted smoking to the vulnerable and disenfranchised schizophrenia population, by proliferating the self-medication hypothesis and research into the ability of nicotine to modulate cognitive function, and thwarted policy efforts to restrict smoking in or near psychiatric facilities [66]. In a way, this could have delayed the advancement of research and ideas from nonindustry researchers and in turn slowed broader public health changes. Despite the various viewpoints, the fact remains that the unlikely dyadic relationship between the tobacco industry and the medical researcher helped forward a significant amount of research pertaining to smoking and schizophrenia, for the better or for the worse.

### 2.7. Despite Barriers, Smoking Cessation in Schizophrenia Is Realistic

Patients with schizophrenia face additional barriers in reaching smoking cessation compared to patients without. One study suggested that dopamine receptor D2 antagonists attenuate the reward effects of nicotine [72]. This pharmacological interaction could partly explain why patients with schizophrenia smoke more cigarettes and inhale more deeply than others [5, 6]. There is also evidence that there are increased connections from the nucleus accumbens to the middle temporal gyrus and precuneus during episodes of cigarette craving. These structures are part of the default mode network, which are interlacing regions of the brain that activate when individuals focus inwardly rather than on the external world [73]. This could suggest that schizophrenia patients feel a stronger subjective attachment to cigarettes than people with schizophrenia.

A recent meta-analysis reported the prevalence of smoking cessation in participants with schizophrenia to be 14.5%, compared to 23.1% in healthy controls and 19.6% in those with other psychiatric illnesses [74]. However, the comparatively low prevalence of smoking cessation in schizophrenia may be representative of ineffective smoking cessation policies or strategies in that population, rather than corroborative of neurobiological theories as to why the prevalence is lower. Providers may be less likely to engage schizophrenia patients in smoking cessation discussion for fear of worsening schizophrenia symptoms, despite a vast body of evidence that refutes this concern [47, 75].

## 3. Conclusions

Cigarette smoking is a major modifiable risk factor for adversely health outcomes in individuals with schizophrenia. Besides clinical symptoms, tobacco smoking additionally carries a heavy financial burden. There are many hypotheses attempting to explain underlying factors driving high smoking rates in patients with schizophrenia. One is a self-medicative hypothesis, another is a theory that there is shared genetic propensity in smoking and development of schizophrenia, and the third is smoking itself may play a role in the development of schizophrenia.

Evidence suggests pharmacotherapy has a low but measurable effectiveness at attaining smoking cessation schizophrenia patients; however, current evidence is limited by lack of studies that adjust for active psychiatric symptoms or polysubstance use. While smoking cessation should be encouraged, it is important to note that nearly all the evidence that attests to the safety of smoking cessation and drugs such as varenicline, bupropion, and NRT in schizophrenia patients was in patients in medical and psychiatric remission. Side effects of both medications should be monitored during smoking cessation. The existing literature suggests that smoking cessation drugs and attempts do not induce moderate to severe psychiatric symptoms in schizophrenia and that cessation is associated with mortality benefits despite weight gain. Smoking additionally has a significant effect on the pharmacological management of schizophrenia, as metabolism clozapine and olanzapine is increased due to induction of CYP1A2 activity.

Ultimately, patients with schizophrenia face additional barriers when trying to cease smoking. Despite various neurobiological theories attempting to explain lower prevalence of smoking cessation in patients with schizophrenia, reasons for high smoking rates and difficulties with smoking cessation are not fully understood. Effective approaches to address smoking prevalence and smoking cessation are required to lessen the biopsychosocial burden experienced in this population.
